# A comparative study of Pap smear findings among HIV positive and negative women at Government Hospital of Thoracic Medicine (GHTM), Tambaram

**DOI:** 10.1186/1471-2334-12-S1-P35

**Published:** 2012-05-04

**Authors:** GV Seethalakshmi, D Shoba, KR Mohan, C Sourabh, G Manoharan, C Chandrasekar

**Affiliations:** 1 Government Hospital of Thoracic Medicine (GHTM), Tambaram, Chennai, Tamil Nadu, India; 2 International Training and Education Centre on Health (I-TECH India/AroGyaan), Chennai, Tamil Nadu, India; 3 Arignar Anna Memorial Cancer Institute and Hospital, Kanchipuram, Tamil Nadu, India

## Background

Cancer cervix is a major health problem in India, accounting for 26.1-43.8% of all cancers in Indian women. Dysplasia on Pap smear has been reported in 15-40% of HIV positive women which is 10-11 times higher than those observed among HIV negative women. Patients with persistent inflammatory Pap smears harbor high proportion of HPV infection and cervical intraepithelial neoplasia (20.9%). The purpose of this present study was to compare Pap smear findings in HIV-infected and uninfected women at GHTM and to correlate the Pap smear abnormalities among HIV positive women with their immune status.

## Methodology

All women (18-45 years of age), admitted at GHTM, between July-Sept. 2010, consented were selected and Pap smear was performed. Smears were reported by a pathologist blinded to the HIV status of patients. Results were analyzed by using PASW 18 version.

## Results

Total 300 samples were collected in which 204 were HIV positive and 96 were HIV negative. HIV positive women (58.8%) had more Pap smear abnormalities such as cervical dysplasia (3.92%), inflammatory smear (51.96%), infection (2.94%) (p=0.010) when compared with HIV negative women (43.75). Among HIV positives 63.93% had CD4 count less than 250 (p=0.048), and 64.3% had coexisting opportunistic infection (p=0.04). On speculum examination, cervicitis was present in 63.3% of HIV positive women with abnormal pap smears (p=0.004).

## Conclusion

This study showed a high percentage of inflammatory Pap smears (51.96%) among HIV positives. Hence these patients will require a follow up Pap smear and colposcopy and biopsy if inflammation is persistent to exclude cervical cancer.

